# Draft Genome Sequence of a Potential Plant Growth-Promoting Rhizobacterium, *Pseudomonas* sp. Strain CK-NBRI-02

**DOI:** 10.1128/MRA.01113-19

**Published:** 2019-10-24

**Authors:** Mayank Gupta, Puneet Singh Chauhan, Sudhir K. Sopory, Sneh L. Singla-Pareek, Ashwani Pareek, Nidhi Adlakha, Charanpreet Kaur

**Affiliations:** a International Centre for Genetic Engineering and Biotechnology, New Delhi, India; b Microbial Technologies Division, CSIR-National Botanical Research Institute, Lucknow, Uttar Pradesh, India; c School of Life Sciences, Jawaharlal Nehru University, New Delhi, India; d School of Biotechnology, Jawaharlal Nehru University, New Delhi, India; University of Arizona

## Abstract

*Pseudomonas* sp. strain CK-NBRI-02 is a potential plant growth-promoting Gram-negative rhizobacterium isolated from the rhizosphere of maize plants growing in fields in Srinagar, Jammu, and Kashmir, India. Here, we report a 5.25-Mb draft assembly of the genome sequence of *Pseudomonas* sp. strain CK-NBRI-02 with an average G+C content of 62.47%.

## ANNOUNCEMENT

*Pseudomonas* sp. strain CK-NBRI-02, a potential plant growth-promoting microbial strain, was isolated from the rhizosphere of maize plants growing in agricultural soil in Srinagar, Jammu, and Kashmir, India. In order to gain an understanding of the genetic architecture of this strain, we performed Illumina-based whole-genome sequencing of *Pseudomonas* sp. strain CK-NBRI-02.

For the isolation of bacteria, 1 g of rhizospheric soil (∼1-mm-thick region of soil surrounding plant roots) was suspended in 9 ml sterile distilled water. Serial dilutions (10^−2^ to 10^−6^) of the suspension were then plated onto nutrient agar (NA) followed by incubation at 28°C for 24 h. Bacterial colonies so obtained were subsequently subcultured and purified by streaking onto fresh NA plates and were ultimately preserved as glycerol stock. Initial 16S rRNA gene sequence analysis using the 27F (5′-AGAGTTTGATCMTGGCTCAG-3′) and 1492R (5′-TACGGYTACCTTGTTACGACTT-3′) primers revealed close sequence similarity of *Pseudomonas* sp. strain CK-NBRI-02 with *Pseudomonas* sp. strain HMSC08G10, as deduced using BLAST search (1). For whole-genome sequencing studies, the strain was restreaked onto an NA plate, and a single colony was inoculated in liquid broth for genomic DNA isolation using the GenElute bacterial genomic DNA kit (Sigma-Aldrich). The concentration and purity of genomic DNA were determined using a Qubit 2.0 fluorometer and with agarose gel electrophoresis. Subsequently, a library was prepared using the NEBNext Ultra DNA library prep kit for Illumina (New England Biolabs, Ipswich, MA) as per the manufacturer’s instructions.

The genome sequence of *Pseudomonas* sp. strain CK-NBRI-02 was obtained via paired-end sequencing on the Illumina HiSeq 2500 platform. Cutadapt 1.14 (2) was used to remove Illumina adapter sequences. All low-quality (Q < 30) data were filtered out using Sickle 1.33 (3). Reads of less than 30 bases were discarded. Duplicate reads were removed using FastUniq 1.1 (4). After filtering 425.85 Mb of raw data at approximately 80-fold coverage, we obtained 376.91 Mb of clean paired-end reads with a 62.47% average DNA G+C content. The reads were assembled using the Velvet 1.2.10 (5) and MaSuRCA 2.2.1 (6) tools separately and, thereafter, merged using Graph Accordance Assembly (GAA) 1.0 (7). The final draft genome of *Pseudomonas* sp. strain CK-NBRI-02 was obtained after scaffolding of the merged assembled FASTA sequences using PAGIT 1 (8), and the total filtered reads were assembled into 43 scaffolds containing a total of 5,253,286 bp. The scaffold *N*_50_ length was 2,42,646 bp. The longest and shortest scaffolds were, respectively, 4,84,981 bp and 631 bp long. The average length of the scaffolds was 122,169 bp.

We used the standalone Prokaryotic Genome Annotation Pipeline (PGAP, 2019-08-01.build3919) (9) for the annotation of the final draft genome of *Pseudomonas* sp. strain CK-NBRI-02. A total of 4,888 genes comprising 4,809 coding sequences (CDS), 76 pseudogenes, and 79 RNA genes, including 10 rRNA (5 5S rRNAs, 2 16S rRNAs, and 3 23S rRNAs) and 65 tRNA genes, were predicted. Default parameters were used for all the software unless otherwise specified.

Detailed investigations of the isolated strain and its comparison with known plant growth-promoting strains will help in delineating the evolutionary relationships and biological relevance of this strain.

### Data availability.

This whole-genome shotgun project has been deposited at DDBJ/ENA/GenBank under the accession number VSJH00000000. The version described in this paper is version VSJH01000000. The BioProject accession number is PRJNA478291.
